# Influencing factors of participation in and satisfaction with elderly health checkups: a cross-sectional study

**DOI:** 10.3389/fpubh.2023.1104438

**Published:** 2023-04-27

**Authors:** Ying-Jen Chen, Chiou-Fen Lin, Jie Feng, Huei-Ling Chiu

**Affiliations:** ^1^Division of Geriatrics and General Internal Medicine, Department of Internal Medicine, Chang Gung Memorial Hospital, Taoyuan, Taiwan; ^2^School of Medicine, College of Medicine, Chang Gung University, Taoyuan, Taiwan; ^3^School of Gerontology and Long-Term Care, College of Nursing, Taipei Medical University, Taipei, Taiwan; ^4^Department of Global Public Health, Karolinska Institutet, Stockholm, Sweden; ^5^International Ph.D. Program in Gerontology and Long-Term Care, College of Nursing, Taipei Medical University, Taipei, Taiwan

**Keywords:** preventive health service, sociodemographic, chronic disease, subjective satisfaction, participation rate

## Abstract

**Background:**

Attending health checkups as a primary prevention strategy benefits older adults in facilitating the identification of health issues and risk factors for disease. Little is known about factors influencing participation in and satisfaction with a free annual elderly health checkup program (EHCP) in Taiwan. This study aimed to extend current knowledge related to the uptake of this service and individuals' views of the service.

**Methods:**

This was a cross-sectional study using a telephone interview survey method to compare influencing factors and satisfaction between participants and non-participants of an EHCP. The individuals involved were older adults in Taipei, Taiwan. The random sampling method included 1,100 people, 550 older adults who had participated in the EHCP within the last 3 years, and 550 older adults who had not. A questionnaire containing personal characteristics and satisfaction with the EHCP was used. Independent *t*-test and Pearson's Chi-squared test were used to evaluate differences between the two groups. Associations between individual characteristics and health checkup attendance were estimated using log-binomial models.

**Results:**

Results showed that 51.64% of participants reported being satisfied with the checkups; however, only 41.09% of non-participants were satisfied. In the association analysis, age, educational level, chronic diseases, and subjective satisfaction were related to older persons' participation. Furthermore, having a stroke was associated with a higher attendance rate [prevalence ratio: 1.49; 95% confidence interval: (1.13, 1.96)].

**Conclusions:**

The EHCP had a high proportion of satisfaction among participants, but the proportion was low among non-participants. Several factors were associated with participation and might lead to unequal healthcare service uptake. Health checkups need to increase among people at a young age, those with low educational backgrounds, and those without chronic diseases.

## 1. Introduction

Population aging in Taiwan is already occurring. Taiwan became an “aged society” in 2018, with more than 14% of the population ≥65 years old (1). The aged population is rapidly increasing and is expected to reach 20% or higher by 2025, which could make Taiwan become a “super-aged society” (1). As a result of changing demographics, chronic diseases are becoming more widespread. Over 85% of older adults in Taiwan suffer from at least one chronic condition (2). Chronic diseases such as cancer, heart disease, hypertension, diabetes, and cerebrovascular disease are already leading causes of death in Taiwan (3).

Health checkups help identify health issues and risk factors for disease or provide reassurance to people without a specific medical indication (4, 5). They may help address persistent health inequalities, expanding numbers of people living with long-term diseases, and the growing needs of an aging population (6). Routine health checkups are essential for reducing healthcare costs related to chronic diseases (7, 8). It is necessary to identify risk factors for conditions such as heart disease, diabetes, and stroke (7, 8). People with routine health checkups are more likely to get an early diagnosis if they develop a medical condition, leading to better outcomes and longer lifespans (9). Health checkups may also be referred to as medical screening, preventive or pre-symptomatic tests, or preventative examinations (10). They may be a cost-effective way of addressing the causes of illnesses before they develop into severe long-term conditions (6).

Despite the potential necessity and benefits of such health checkups, it is well-recognized that their uptake is largely sub-optimal. The Taipei City government has provided annual elderly health checkups (EHCP) at no cost for all seniors in Taipei since 2014. The EHCP cover physical assessments, depression screening, cognitive function assessments, a routine urine assay, a routine blood assay, biochemical assays (for albumin, globulin, uric acid, and blood urea nitrogen), a fecal occult blood test, and a fecal immunochemical test. Between 37,000 and 46,000 services are provided yearly (11). However, only a small group of people (fewer than 10% of older adults) participate in health checkups every year, and several provided services are not fully utilized (11). Differential uptake may make health inequities worse (12). Consequently, knowledge of the factors influencing health checkup attendance is fundamental if current services can be adequately changed to mitigate such inequities.

A few national and international studies have investigated factors influencing the decision to get a health checkup. Previous research revealed that sociodemographic characteristics, lifestyle factors, and a person's medical history were significant factors influencing the decision to get a checkup (13, 14). Another factor is an individual's health beliefs, based on values ascribed to health and a belief in the efficacy of health checkups (6, 15). However, little is known about factors that influence attendance of annual government-provided free health checkups. As a unique context, associations could differ. Furthermore, to target deficiencies in the EHCP, an investigation targeting population satisfaction is necessary.

This study aimed to extend current knowledge relating to the uptake of EHCP services and individuals' views of this service and thus contribute to improving healthcare service delivery and the further development of government policies. In particular, our study sought to address two questions:

1. What are the factors influencing attendance of EHCPs among senior citizens in Taiwan?
2. To what extent do EHCPs satisfy seniors in Taiwan?

## 2. Materials and methods

### 2.1. Study design, setting, and participants

This comparative cross-sectional study used a telephone interview survey method in Taipei, Taiwan to compare factors influencing whether to participate in an EHCP and satisfaction with the program. The total population of Taipei City was 2,645,041 in December 2019, of which 477,944 (18%) persons were ≥65 years. The EHCP especially targets Taipei City residents aged ≥65 years and indigenous people living anywhere in Taipei who are aged ≥55 years. In this study, data on participants who were eligible to participate in the EHCP (Taipei City residents aged ≥65 years and indigenous people living anywhere in Taipei who are aged ≥55 years) were retrieved from a registry of senior citizens at the Department of Health, Taipei City government. A random sampling method included 1,100 people, 550 older adults who had participated in an EHCP within the last 3 years and 550 older adults who had not. For each group, an interview list (including 2,000 people) was generated from a table of random numbers by a computer. We conducted telephone interviews according to the lists until 550 older adults in each group had been interviewed. The sample size was calculated based on the recommendations of Streiner et al., who suggested a sample of 5–20 participants for each questionnaire item (16). For this study, it was decided to include a minimum of 20 participants per question, resulting in a sample size of at least 160 participants; as for the suggestion by Comrey and Lee, the sample size of 500 means “very good” (17).

### 2.2. Measures

The questionnaire administered in the present study (Questionnaire on Publicity for Elderly Health Checkups) contains influencing factors and satisfaction regarding publicity and implementation of the EHCP. It was a self-developed questionnaire, developed by a focus group discussion of six medical professionals, revised according to suggestions from another six professionals, pilot-tested, and tested for reliability and validity. It had good validity (with a content validity index of 0.988) and reliability (Cronbach's α of 0.730).

The questionnaire consisted of two parts: (1) personal characteristics and (2) satisfaction with the EHCP. Personal characteristics were sociodemographic data and medical information, including age, sex, educational level, and health condition. As for citizens' satisfaction with the EHCP, the survey included eight parts: (1) publicity methods; (2) overall appointment methods; (3) appointment by telephone; (4) appointment online; (5) appointment on site; (6) health checkup items; (7) number of services provided; and (8) follow-up services. Participants were asked, “To what extent are you satisfied with …?”. For example, to what extent are you satisfied with the publicity methods of the EHCP. Satisfaction was measured by a Likert Scale, scored from 1 (very unsatisfied) to 5 (very satisfied).

Interviewers were students from Taipei Medical University with medical or healthcare backgrounds. In total, 11 interviewers were selected and were well-trained before the interview. All interviewers participated in a training workshop and had undergone rehearsals with the researchers. The research team also prepared an interview draft form to ensure consistency among interviewers.

Approval from Taipei Medical University Joint Institutional Review Board (no. N201907066) was granted. Potential participants were verbally informed of the study's aim and process. Telephone interviews began after receiving participants' verbal consent. In addition, all participants were informed of their right to refuse to answer the questions and end the interview at any time.

### 2.3. Statistical analysis

We used STATA statistical software version 16.1 (StataCorp, College Station, TX, USA) for all statistical analyses and compared demographic data and health conditions (whether participants had chronic diseases or multimorbidity) between the two groups of participants. Study data were examined for outliers, normality, and missing data. The basic information of participants was summarized using descriptive statistics. Continuous variables were transformed or categorized when necessary. Categorical variables were reported as numbers and proportions for each category. The overall satisfaction score for each person was calculated as the average score of the Likert Scale. An independent *t*-test was used to test for the difference of overall satisfaction score between two study groups. Pearson's Chi-squared test or Fisher's exact tests (whether appropriate) were used to explore the differences in individual characteristics between the two groups. Associations between individual characteristics and EHCP participation were estimated with log-binomial models. If the binomial models failed to converge, Poisson regression models with a robust error variance were used instead. All reported *p*-values were two-sided. Statistical significance was defined as *p*<0.05.

## 3. Results

### 3.1. Sociodemographic characteristics of participants

In total, 1,100 individuals were involved in this study. More than half (58.5%) of participants were female, and most were retired (89.27%). Participants were predominantly aged 66–75 (55.09%) or 75–85 years (33.82%). Other demographic and socioeconomic characteristics are presented in Table 1.

**Table 1:** Sociodemographic characteristics of individuals.

**Sociodemographic characteristic**	**Frequency (*N* = 1,100)**	**Percentage (%)**
**Sex**		
Male	449	40.80%
Female	643	58.50%
Missing data	8	0.70%
**Age (years)**		
55–65	52	4.73%
66–75	606	55.09%
75–85	372	33.82%
>85	65	5.91%
Missing data	5	0.45%
**Educational level**		
Low	222	20.18%
Middle	685	62.27%
High	182	16.55%
Missing data	11	1.00%
**Marital status**		
Married	968	88.00%
Unmarried	76	6.91%
Missing data	56	5.09%
**Occupation (current)**		
Retired	982	89.27%
Still working	110	10.00%
Missing data	8	0.73%

Educational level: Low (Illiterate or elementary school educated); Middle (Junior or Senior high school educated); High (University and above).

### 3.2. Satisfaction with the EHCP

As for citizens' satisfaction with the EHCP, the survey included eight parts: (1) publicity methods; (2) overall appointment methods; (3) appointment by telephone; (4) appointment online; (5) appointment on-site; (6) health checkup items; (7) number of services provided; and (8) follow-up services. In total, 777 participants completely answered this part, and details are given in Table 2. Compared to participants of the EHCP, more non-participants were dissatisfied with the publicity methods, health checkup items, number of services provided, and health checkup process. We also calculated the overall satisfaction score and found that those who had used the EHCP in the past 3 years had a statistically significantly higher level of satisfaction (Mean = 3.58, SD = 0.03) than those who had not (Mean = 3.40, SD = 0.03, *t* = 4.6554, df = 777, *p* < 0.001). We considered being satisfied with the ECHP as having an overall score of more than 3 and being dissatisfied as having a score of <3. Additionally, 284 participants (51.64% of total 550) who had used the EHCP in the past 3 years reported being satisfied; however, among those who had not used the EHCP in the past 3 years, only 226 participants (41.09% of total 550) were satisfied.

**Table 2:** Individuals' satisfaction with the elderly health checkup program (EHCP).

**Items**	**Very dissatisfied**		**Dissatisfied**		**Neutral**		**Satisfied**		**Ver y satisfied**		**Median (IQR)**
	***n***	**%**	***n***	**%**	***n***	**%**	***n***	**%**	***n***	**%**	
	Group 1 *N* = 387
Publicity methods	0	0.00%	10	2.58%	169	43.67%	160	41.34%	48	12.40%	4 (3–4)
Overall appointment methods	1	0.26%	11	2.84%	179	46.25%	155	40.05%	41	10.59%	3 (3–4)
Appointment by telephone	0	0.00%	10	2.58%	191	49.35%	138	35.66%	48	12.40%	3 (3–4)
Appointment online	2	0.52%	30	7.75%	216	55.81%	100	25.84%	39	10.08%	3 (3–4)
Appointment on-site	2	0.52%	17	4.39%	205	52.97%	105	27.13%	58	14.99%	3 (3–4)
Health checkup items	0	0.00%	17	4.39%	140	36.18%	164	42.38%	66	17.05%	4 (3–4)
Number of services provided	1	0.26%	43	11.11%	151	39.02%	133	34.37%	59	15.25%	3 (3–4)
Health checkup process	0	0.00%	5	1.29%	159	41.09%	154	39.79%	69	17.83%	4 (3–4)
	Group 2 *N* = 392
Publicity methods	2	0.51%	29	7.40%	191	48.72%	140	35.71%	30	7.65%	3 (3–4)
Overall appointment methods	0	0.00%	9	2.30%	222	56.63%	135	34.44%	26	6.63%	3 (3–4)
Appointment by telephone	0	0.00%	7	1.79%	254	64.80%	97	24.74%	34	8.67%	3 (3–4)
Appointment online	3	0.77%	28	7.14%	283	72.19%	55	14.03%	23	5.87%	3 (3–3)
Appointment on-site	2	0.51%	14	3.57%	245	62.50%	108	27.55%	23	5.87%	3 (3–4)
Health checkup items	0	0.00%	23	5.87%	193	49.23%	139	35.46%	37	9.44%	3 (3–4)
Number of services provided	3	0.77%	45	11.48%	208	53.06%	94	23.98%	42	10.71%	3 (3–4)
Health checkup process	0	0.00%	9	2.30%	203	51.79%	126	32.14%	54	13.78%	3 (3–4)

IQR, interquartile range.

### 3.3. Factors influencing participation in the EHCP

Among the 1,100 participants, 550 had experienced the EHCP in the past 3 years, and the others had not. Results of the bivariate analysis (Table 3) indicated significant differences in some variables between participants and non-participants of the EHCP. Participants tended to be aged 76–85 years, have a low educational level, have more than two chronic diseases, have diabetes or stroke, and be satisfied with the EHCP. No significant differences were reported in sex, marital status, hypertension, heart disease, or hyperlipidemia between participants and non-participants of the EHCP.

**Table 3:** Bivariate correlates of participation in the elderly health checkup program (EHCP) among the elderly.

**Variable**	**Group 1: participated (*****N*** = **550)**		**Group 2: did not participate (*****N*** = **550)**		**χ^2^**	***p*-value**
	***n***	**%**	***n***	**%**		
**Age (years)**						
55–65	15	2.73%	37	6.73%	13.74	0.003
66–75	295	53.64%	311	56.55%		
76–85	205	37.27%	167	30.36%		
>85	31	5.64%	34	6.18%		
Missing data *n* = 5						
**Educational level**						
Low	132	24.00%	90	16.36%	10.92	0.004
Middle	331	60.18%	354	64.36%		
High	81	14.73%	101	18.36%		
Missing data *n* = 11						
**Number of chronic diseases**						
≤ 2	472	85.82%	500	90.91%	6.93	0.008
More than two	78	14.18%	50	9.09%		
**Diabetes**						
Yes	147	26.73%	116	21.09%	4.80	0.028
No	403	73.27%	434	78.91%		
**Stroke (ever)**						
Yes	14	2.55%	5	0.91%	4.34	0.037
No	536	97.45%	545	99.09%		
**Satisfied**						
Yes	284	51.64%	226	41.09%	14.73	0.001
No	21	3.82%	50	9.10%		
Neutral	82	14.91%	116	21.09%		
Missing data *n* = 320						

^*^Only significant correlates are presented.

Associations between personal characteristics and EHCP participation were investigated using log-binomial models. Six factors were identified to be related to participation (Table 4). Individuals aged 55–65 years (they were indigenous people) were less likely than those aged 66–75 years to have participated the EHCP [Prevalence ratio (PR) with 95% confidence interval (95% CI): 0.59 (0.38, 0.92)]. Individuals aged 76–85 years, on the other hand, were more likely to have participated the EHCP [PR (95% CI): 1.13 (1.00, 1.28)]. Seniors with a low educational level were more likely than seniors with a middle educational level to have participated the EHCP [PR (95% CI): 1.23 (1.07, 1.40)]. Individuals who were neutral or dissatisfied with the program were less likely to have participated in the EHCP [PR (95% CI): 0.74 (0.62, 0.89) and 0.75 (0.58, 0.97), respectively]. Individuals with more than two chronic conditions were also more likely to have participated the EHCP than those who did not have those conditions [PR (95% CI): 1.25 (1.08, 1.46)]. We also looked at the relationship between having a specific chronic disease and participating the EHCP. Patients who had had a stroke or had diabetes were more likely to have participated than those who did not [PR (95% CI): 1.49 (1.13, 1.96) and 1.16 (1.02, 1.32), respectively].

**Table 4:** Results of log-binomial models examing factors associated with participation in the EHCP among the elderly.

**Variables**	**Reference category**	**PR**	**95% CI**		***p*-value**
			**Lower**	**Upper**	
Age	66–75				
55–65		0.59	0.38	0.92	0.018
76–85		1.13	1.00	1.28	0.048
>85		0.98	0.75	1.28	0.881
Educational levels	Middle				
Low		1.23	1.07	1.40	0.002
High		0.92	0.77	1.10	0.370
Satisfied	Yes				
No		0.75	0.58	0.97	0.031
Neutral		0.74	0.62	0.89	0.001
Number of chronic diseases	≤2				
More than two		1.25	1.08	1.46	0.004
Stroke (ever)	No				
Yes		1.49	1.13	1.96	0.005
Diabetes	No				
Yes		1.16	1.02	1.32	0.023

## 4. Discussion

This study investigated the EHCP in Taiwan in terms of satisfaction and determinants of participation. We found that the participants of the EHCP were more satisfied with it, and non-participants were mainly dissatisfied with the publicity methods, health checkup items, number of services provided, and the health checkup process. Additionally, satisfaction was found to be positively associated with healthcare service utilization, as a prior study also showed (18). As for satisfaction, there are still some aspects that could be improved. The primary publicity method is sending ads to eligible citizens by mail, which is a traditional way. It takes time to arrive, and the ad in the mailbox can easily be ignored. However, this method works well for targeting seniors, since they are comfortable engaging through this channel and still use it regularly. Other methods, such as making a phone call or advertising on the Internet, could also be effective because of the increasing smartphone use among seniors in Taiwan (19). As for health checkup items and processes, a former study suggested that a belief that screening procedures were too complicated to understand was one of the factors that reduced the willingness to participate in health screening in Taiwan (7). Additionally, bad experiences (e.g., a long waiting time) during a previous health checkup negatively influence attendance (7, 20). Every year, the city government offers a limited number of free health checkups for all senior residents and gives priority to more disadvantaged people, including residents who (1) are living alone; (2) live in low-income households or middle-low-income households; (3) have physical or mental disabilities, and (4) are indigenous residents. Other citizens can only competitively book a spot in the second round, which might be the reason that non-participants were dissatisfied with the number of services provided. To improve access to free health services, further study is needed to estimate the number of older adults who need annual health checkups to prevent diseases and provide suggestions to providers to make this free service more accessible.

The absence of sex or marital status differences in participation in the EHCP is interesting. This result differed from previous studies which examined factors predicting the use of other preventive or medical services. Females tend to use preventive and diagnostic services more frequently (21, 22), and the use of health services varies according to one's life stage, with women using the healthcare system more frequently during their childbearing years (23). One methodological reason for this phenomenon could be different definitions of preventative health checkups, including gynecological cancer examinations such as mammography and cervical cancer screening, which are otherwise excluded. Another reason men and women use preventative healthcare services equally today could be that gender-specific initiatives have transformed the general approach to illness and health (23, 24). Other studies indicated that marriage promotes screening attendance, and both a single and divorce status were considered factors that increased barriers to screening (25, 26). One potential explanation for the absence of an impact of the marital status could be that there is an interaction between marital status and sex, but no sex difference was found.

Results of a higher likelihood of EHCP participation by older adults with more than two chronic diseases compared to those without any are consistent with previous findings, as individuals suffering from at least one chronic disease or disorder engaged more in prevention than people without such complaints (24). Additionally, we found that individuals with diabetes or who had had a stroke had a higher probability of participation than those without, which is also in line with previous findings that individuals with diabetes were more likely to attend health checkups (27, 28). Possible explanations might be that (1) chronically diseased individuals who are regularly in contact with health professionals under permanent medical supervision may be easily informed about the EHCP; (2) susceptibility to potential health conditions or comorbidities might increase the likelihood of attending a free preventive health checkup, which is reasonable in the context of primary prevention; and alternatively, (3) annual or more frequent checkups are necessary for persons with a chronic disease, as research indicates that preventative care decreases the prevalence and progression of such conditions (29).

Other potential influencing factors identified in this study were educational level and age. Indigenous residents in Taiwan have a lower life expectancy and suffer more from alcoholism and other poor health outcomes than the general population (30). Despite having priority in participation of the EHCP, compared to individuals aged 66–75 years, they were less likely to attend health checkups. Our finding also indicates that there might still be a disparity in participation in the EHCP between indigenous residents and the general population. Age affects participation in health screening, with older seniors having relatively high participation rates, consistent with earlier research (31, 32). However, participation decreased with a higher education level, as another study suggested (31). This might be because highly educated people are more health-conscious and demand higher quality and quantity of health screening.

This is the first study to examine both participants' and non-participants' satisfaction with the EHCP in Taipei and estimate associations between personal characteristics and participation. Strengths of the present study include the large sample size and the comparative study design, which allowed distinct comparisons. In this study, the measurement (questionnaire for a telephone interview) was tested for reliability and validity, increasing the likelihood of data consistency. Because of the large number and random selection of interview participants, a high external validity of the results for Taiwan can be assumed.

One major methodological limitation of this study is the fact that the study was cross-sectional and lacked causal inferences for the associations we found. In addition, we defined non-participants as those who had participated in the EHCP within the last 3 years, including those who once participated in EHCP but withdrew for some reason and those who had never participated. Therefore, it ignored the differences between these two groups of people. However, this study mainly focuses on the differences between participants and non-participants of the EHCP. A deeper study was needed to further investigate the different opinions of the EHCP among different non-participants Additionally, personal characteristics were self-reported rather than taken from recorded data in the national healthcare system. No validation was carried out using a patient's medical charts. The questionnaire pre-set the personal characteristics and the influencing factors investigated were limited. In the analysis, the estimated associations were not adjusted for potential confounders, unlike other studies investigating predictors. An analytical study design rather than a descriptive study could further estimate associations between personal characteristics and health checkup participation.

## 5. Conclusions

From the present results, it can be concluded that the EHCP had a high proportion of satisfaction among participants, but the proportion was relatively low among non-participants. It is suggested that publicity methods, health checkup items, the number of services provided, and the health checkup process could be further improved. The findings suggest that age, educational level, chronic diseases, and subjective satisfaction were related to older persons' participation in Taipei's annual health checkup program. Preventive screening could benefit older adults by detecting diseases early and help them maintain their health. This study can help policymakers target non-participants or disadvantaged groups in the program and recommend measures to promote their uptake.

## Data availability statement

The raw data supporting the conclusions of this article will be made available by the authors, without undue reservation.

## Ethics statement

The studies involving human participants were reviewed and approved by Taipei Medical University-Joint Institutional Review Board (N201907066). Written informed consent for participation was not required for this study in accordance with the national legislation and the institutional requirements.

## Author contributions

Y-JC, C-FL, and H-LC contributed to conception and design of the study. C-FL, JF, and H-LC organized the database. JF performed the statistical analysis. Y-JC and C-FL wrote the first draft of the manuscript. Y-JC, C-FL, JF, and H-LC wrote sections of the manuscript. All authors contributed to manuscript revision, read, and approved the submitted version.

## References

[1] National Development Council. Population Projections for the R.O.C. (Taiwan). Taipei, Taiwan: National Development Council (2022). Available online at: https://pop-proj.ndc.gov.tw/main_en/index.aspx (accessed January 25, 2023).

[2] Health Promotion Administration. National Health Interview Survey (NHIS). Taiwan: Ministry of Health and Welfare (2017).

[3] Ministry of Health and Welfare. 2020 Cause of Death Statistics. Taiwan (2021).

[4] Stol YH Asscher ECA Schermer MHN. Good health checks according to the general public; expectations and criteria: a focus group study. BMC Med Ethics. (2018) 19:64. 10.1186/s12910-018-0301-6 29929500 PMC6013874

[5] Krogsbøll LT Jørgensen KJ Gøtzsche PC. General health checks in adults for reducing morbidity and mortality from disease. Cochrane Database Syst Rev. (2019) 1:Cd009009. 10.1002/14651858.CD009009.pub3 30699470 PMC6353639

[6] Dryden R Williams B McCowan C Themessl-Huber M. What do we know about who does and does not attend general health checks? Findings from a narrative scoping review. BMC Public Health. (2012) 12:723. 10.1186/1471-2458-12-723 22938046 PMC3491052

[7] Chien S-Y Chuang M-C Chen I-P. Why people do not attend health screenings: factors that influence willingness to participate in health screenings for chronic diseases. Int J Environ Res Public Health. (2020) 17:3495. 10.3390/ijerph17103495 32429532 PMC7277138

[8] Office Office of Disease Prevention Health Promotion UPSTF States. U. Guide to clinical preventive services: report of the US Preventive Services Task Force. US Department of Health and Human Services. (1996).

[9] Johns Hopkins Medicine. Routine Screening. (2022). Available online at: https://www.hopkinsmedicine.org/health/treatment-tests-and-therapies/routine-screenings (accessed January 25, 2023).

[10] Stol YH Asscher ECA Schermer MHN. What is a good health check? An interview study of health check providers' views and practices. BMC Medical Ethics. (2017) 18:55. 10.1186/s12910-017-0213-x 28969671 PMC5625608

[11] Ministry of Health Welfare. Statistics Newsletters. (2020). Available online at: https://www-ws.gov.taipei/Download.ashx?u=LzAwMS9VcGxvYWQvNjg0L3JlbGZpbGUvNDcxMDIvODE2MjA4_OC83NTgyMTg5Yy1hZDM3LTQwOTItYjk2Yy03NjBmM2VjMTcxOTYucGRm&n=6KGb56aP57Wx6KiI57Ch6KiKLeiHuuWMl%2BW4guiAgeS6uuWBpeaqouamguazgTEwOTAz_MTIucGRm&icon=.pdf (accessed January 25, 2023).

[12] Hart JT. The inverse care law. Lancet. (1971) 297:405–12. 10.1016/S0140-6736(71)92410-X 4100731

[13] Iwasaki M Otani T Yamamoto S Inoue M Hanaoka T Sobue T. Background characteristics of basic health examination participants: the JPHC study baseline survey. J Epidemiol. (2003) 13:216–25. 10.2188/jea.13.216 12934965 PMC9663412

[14] Yoshida Y Iwasa H Kwon J Furuna T Kim H Yoshida H. Characteristics of non-participants in comprehensive health examinations (“Otasha-kenshin”) among an urban community dwelling elderly: basic research for prevention of the geriatric syndrome and a bed-ridden state. [Nihon Koshu Eisei Zasshi] *Jpn J Public Health*. (2008) 55:221–7. 18536336

[15] Hsu HC Luh DL Chang WC Pan LY. Joint trajectories of multiple health-related behaviors among the elderly. Int J Public Health. (2013) 58:109–20. 10.1007/s00038-012-0358-9 22438083

[16] Streiner DL Norman GR Cairney J. Health Measurement Scales: A Practical Guide to Their Development and Use. Oxford University Press (2014). 10.1093/med/9780199685219.001.000127242256

[17] Comrey AL Lee HB. A First Course in Factor Analysis. New York: Psychology Press (2013). 10.4324/9781315827506

[18] Biondi EA Hall M Leonard MS Pirraglia PA Alverson BK. Association between resource utilization and patient satisfaction at a tertiary care medical center. J Hosp Med. (2016) 11:785–91. 10.1002/jhm.2621 27272894

[19] Chen AN McGaughey RE Zeltmann SM Lu H-K Lee MR. How seniors in Taiwan use information technology: computer and cell phones. Int J Hum Comput Interact. (2018) 34:166–76. 10.1080/10447318.2017.133544534250912

[20] Dickinson JA Pimlott N Grad R Singh H Szafran O Wilson BJ. Screening: when things go wrong. Can Fam Physician. (2018) 64:502–8. 30002025 PMC6042667

[21] Gómez Gómez E. Género, equidad y acceso a los servicios de salud: una aproximación empírica. Rev Panamericana Salud Pública. (2002) 11:327–34. 10.1590/S1020-49892002000500008 12162830

[22] Hoebel J Starker A Jordan S Richter M Lampert T. Determinants of health check attendance in adults: findings from the cross-sectional German Health Update (GEDA) study. BMC Public Health. (2014) 14:913. 10.1186/1471-2458-14-913 25185681 PMC4167266

[23] Alexandraki I. Utilization of preventive care services: does gender matter? J Womens Health. (2012) 21:118–20. 10.1089/jwh.2011.3311 22192003

[24] Brunner-Ziegler S Rieder A Stein KV Koppensteiner R Hoffmann K Dorner TE. Predictors of participation in preventive health examinations in Austria. BMC Public Health. (2013) 13:1138. 10.1186/1471-2458-13-1138 24308610 PMC3866300

[25] Wardle J Miles A Atkin W. Gender differences in utilization of colorectal cancer screening. J Med Screen. (2005) 12:20–7. 10.1258/0969141053279158 15814015

[26] Van Jaarsveld CH Miles A Edwards R Wardle J. Marriage and cancer prevention: does marital status and inviting both spouses together influence colorectal cancer screening participation? J Med Screen. (2006) 13:172–6. 10.1177/096914130601300403 17217605

[27] Park BH Lee BK Ahn J Kim NS Park J Kim Y. Association of participation in health check-ups with risk factors for cardiovascular diseases. J Korean Med Sci. (2021) 36:e19. 10.3346/jkms.2021.36.e19 33463093 PMC7813587

[28] Culica D Rohrer J Ward M Hilsenrath P Pomrehn P. Medical checkups: who does not get them? Am J Public Health. (2002) 92:88–91. 10.2105/AJPH.92.1.88 11772768 PMC1447395

[29] Eyre H Kahn R Robertson RM. Preventing cancer, cardiovascular disease, and diabetes: a common agenda for the American Cancer Society, the American Diabetes Association, and the American Heart Association. Diabetes Care. (2004) 27:1812–24. 15220271 10.2337/diacare.27.7.1812

[30] Juan S-C Awerbuch-Friedlander T Levins R. Ethnic density and mortality: aboriginal population health in Taiwan. Public Health Rev. (2016) 37:11. 10.1186/s40985-016-0028-1 29450053 PMC5810052

[31] Du B Mu Y. The relationship between health changes and community health screening participation among older people. Front Public Health. (2022) 10:870157. 10.3389/fpubh.2022.870157 35570968 PMC9091503

[32] Schülein S Taylor KJ Schriefer D Blettner M Klug SJ. Participation in preventive health check-ups among 19,351 women in Germany. Prev Med Rep. (2017) 6:23–6. 10.1016/j.pmedr.2017.01.011 28210539 PMC5310169

